# Intravenous Tranexamic Acid Has Benefit for Reducing Blood Loss after Open-Wedge High Tibial Osteotomy: A Randomized Controlled Trial

**DOI:** 10.3390/jcm10153272

**Published:** 2021-07-24

**Authors:** Man-Soo Kim, In-Jun Koh, Yong-Gyu Sung, Dong-Chul Park, Won-Jun Ha, Yong In

**Affiliations:** 1 Department of Orthopaedic Surgery, Seoul St. Mary’s Hospital, College of Medicine, The Catholic University of Korea, 222, Banpo-daero, Seocho-gu, Seoul 06591, Korea; kms3779@naver.com (M.-S.K.); ygsung@catholic.ac.kr (Y.-G.S.); dc1225@naver.com (D.-C.P.); nanhan9gin@gmail.com (W.-J.H.); 2 Department of Orthopaedic Surgery, Eunpyeong St. Mary’s Hospital, College of Medicine, The Catholic University of Korea, 1021, Tongil Ro, Eunpyeong-gu, Seoul 03312, Korea; oskoh74@gmail.com

**Keywords:** tranexamic acid, blood, loss, high tibial osteotomy, randomized

## Abstract

(1) Background: the purpose of this study was to investigate the efficacy and safety of intravenous (IV) administration of tranexamic acid (TXA) in patients undergoing medial opening wedge high tibial osteotomy (MOWHTO). (2) Methods: a total of 73 patients were randomly allocated into two groups (TXA group and control group). The primary outcome was total perioperative calculated blood loss after MOWHTO. Secondary outcomes included self-reported pain severity using a 10-point visual analog scale (VAS) and the EuroQol-5 Dimension (EQ-5D) questionnaire. The postoperative allogeneic transfusion rate and wound complications were compared. Deep vein thrombosis (DVT) incidence was compared by conducting DVT computed tomography imaging. (3) Results: the total blood loss after surgery was 470.9 mL in the TXA group and 739.3 mL in the control group, showing a significant difference (*p* < 0.001). There were no differences in pain VAS scores between the two groups (all *p* > 0.05). No difference in preoperative EQ-5D scores for any items existed between the two groups. No transfusion was performed in either group. There was no difference in DVT incidence or the rate of wound complications between the two groups. (4) Conclusion: in patients undergoing MOWHTO, IV TXA reduces total blood loss and drainage amount. However, no additional benefits in clinical outcomes, transfusion rate, or wound complications were apparent, with similar DVT incidence rates.

## 1. Introduction

High tibial osteotomy (HTO) is an established and proven surgical method for medial compartmental OA with varus deformity [1,2]. Among HTO techniques, medial opening wedge HTO (MOWHTO) has become more popular than closing wedge HTO in recent years because of the simplicity of surgery [3,4,5]. However, in the case of MOWHTO, major bleeding can occur because the medullary cavity is opened during the osteotomy [6], and only subcutaneous tissue and skin prevent postoperative bleeding from occurring [7,8]. Rates of wound complications, such as hematoma, wound dehiscence, or superficial soft-tissue infection have been reported to be as high as 5% to 10% among MOWHTO patients [9,10,11,12]. In addition, blood transfusions may be necessary due to excessive bleeding [13,14]. Allogenic transfusion is also closely correlated with an increased risk of complications such as blood–bone infections and transfusion-related reactions [15,16]. Therefore, an effective blood management strategy is essential in concert with MOWHTO.

Tranexamic acid (TXA) is well known to reduce bleeding and blood transfusions in a variety of orthopedic procedures [17]. TXA is an antifibrinolytic drug and a synthetic derivative of the amino acid lysine. It acts as a competitive inhibitor of plasminogen activation, preventing fibrinolysis [17]. Several studies, including randomized trials (RCTs) and meta-analyses, have shown that intravenous (IV) TXA can reduce postoperative bleeding and the frequency of transfusions after orthopedic surgery without venous thromboembolic events (VTEs) or increased hospital costs [18,19,20,21].

However, data supporting the routine use of TXA in MOWHTO with respect to the administration method and adverse effects are insufficient in comparison with available data regarding arthroplasty [5,7,8,10,14]. Most existing studies are retrospective [5,8,10,14] and no RCT has clearly assessed the incidence of deep vein thrombosis (DVT) following MOWHTO when using TXA [7]. The purpose of this study was therefore to investigate the efficacy and safety of IV administration of TXA in patients undergoing MOWHTO. The hypothesis of this study was that patients who received IV TXA treatment would have significantly less postoperative bleeding and less pain in the early phase of rehabilitation as compared with those who did not receive IA TXA treatment, without an increased risk of DVT.

## 2. Methods

**Figure 1:**
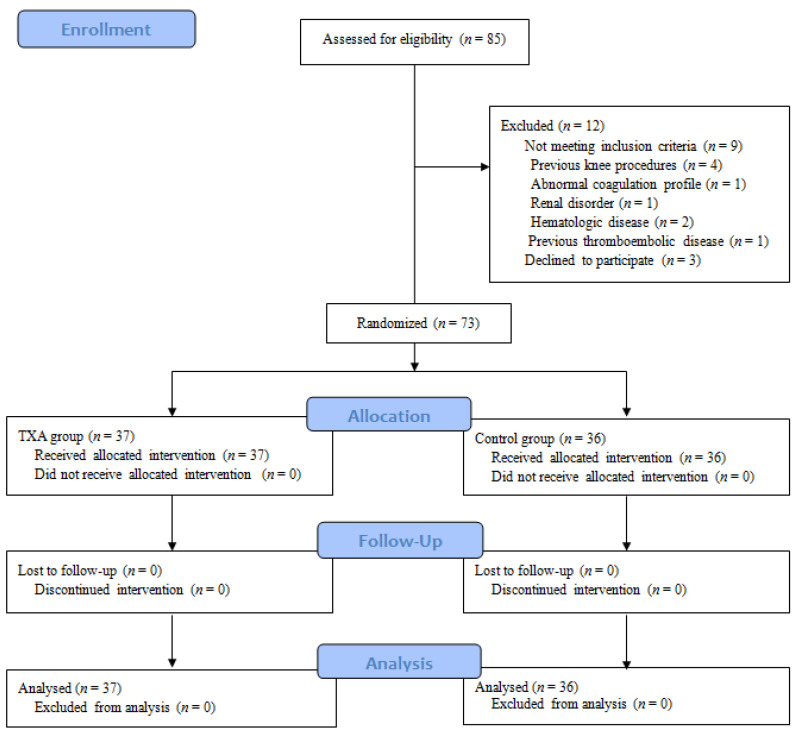
Consort flowchart of study enrollment.

The study was approved by the institutional review board of our hospital (KC19MESI0070, April 2019) and registered at ClinicalTrials.gov (NCT04653623). All patients provided informed consent for their inclusion. Patients who were scheduled for MOWHTO at our institution were screened prospectively between October 2019 and January 2021. The inclusion criterion was MOWHTO performed for medial compartment OA with varus deformity, while the exclusion criteria were procedures performed for reasons other than medial compartment OA (e.g., osteonecrosis or traumatic OA), a history of knee surgery on the affected knee, coagulation disorder (preoperative platelet count < 150,000/mm^3^, INR [(international normalized ratio) >1.4 or prolonged partial thromboplastin time of >1.4 times the normal amount], renal disorder or insufficiency, hematologic disorders (e.g., hematopoietic, hemorrhagic, or thrombogenic disease), a history of arterial or venous thromboembolic disease, and refusal to participate in the study. Among the patients who underwent surgery, patients taking antiplatelet or anticoagulant were referred to the department prescribing the drug so that the drug could be properly adjusted and stopped prior to surgery. A total of 85 patients underwent MOWHTO, and 12 patients were excluded for the following reasons: previous knee procedures on the same side (*n* = 4), abnormal coagulation profile (*n* = 1), renal disorder (*n* = 1), hematologic disorder (*n* = 2), previous thromboembolic disease (*n* = 1), and refusal to participate in this study (*n* = 3). Thus, a total of 73 patients were included in this study and were randomized into two groups (Figure 1).

Patients were randomly assigned to the TXA group (IV administration of TXA) and control group (no administration of TXA) using a computer-based block randomization method. Patient assignments were stored in an opaque sealed bag with serial numbers. Prior to each operation, an independent orthopedic surgeon who did not participate in the surgery opened the bag, and an anesthesiologist who was not involved otherwise in patient treatment performed the procedure. The patients, surgeons, and medical staff involved in treatment and evaluation were blinded to the group assignments during the study period.

### 2.1. Surgical Technique

Correction angles were determined in all patients during preoperative planning via the Dugdale method using weight-bearing, full-length hip–ankle radiographs [22]. The correction angle was calculated using the angle formed by two lines in the preoperative plan, the first line of which ranged from the center of the hip to the so-called Fujisawa point [23], located 62.5% of the width of the tibia in the tibia plateau, and the second line of which ranged from the Fujisawa point on the tibial plateau to the center of the ankle joint [24].

All surgical procedures were performed by two surgeons under general anesthesia with a tourniquet inflated to 300 mmHg. Prior to MOWHTO, arthroscopic procedures were performed in all patients. A longitudinal incision measuring 8 cm in size was applied to the medial side of the proximal tibia. The pes anserinus was identified and released on the medial side of the proximal tibia. The distal portion of the superficial medial collateral ligament was detached from the tibia using a small elevator. Bi-planar MOWHTO was performed according to a technique developed by the Arbeitsgemeinschaft für Osteosynthesefragen knee expert group [25]. A spreader was inserted into the osteotomy site and the osteotomy site was opened gradually to the planned angle. The opening angle was identified from the angle scale on the spreader. The osteotomy site was fixed with a locking plate (Tomofix; Synthes, Solothurn, Switzerland) following appropriate correction. The tourniquet was deflated after plate and screw fixation to cauterize bleeding vessels and restore hemostasis. A suction drain was placed subcutaneously between the plate and skin, and wound closure was performed with a 2-octyl cyanoacrylate adhesive (Dermabond™; Ethicon, Bridgewater, NJ, USA) and self-adhesive polyester mesh (Prineo™; Ethicon, Bridgewater, NJ, USA). The same postoperative rehabilitation program was adopted in both groups. Quadriceps-setting exercises and continuous passive motion began on the first postoperative day. Partial weight-bearing crutch ambulation was allowed for up to six weeks after surgery; then, full weight-bearing ambulation was allowed beginning at six weeks after surgery.

Two grams of TXA (500 mg/5 mL ampoule; Shinpoong, Seoul, Korea) was intravenously injected 10 min before tourniquet inflation, and the same amount was repeatedly injected three hours later [8]. All drains were clamped for three hours. The control group did not receive any such solution.

Mechanical prophylaxis using compression stockings was routinely conducted to prevent DVT; however, no pharmacologic intervention for VTE prophylaxis was adopted. Ice bag placement was also performed routinely during the hospitalization period. The suction drain was removed on the third day after surgery. All patients were discharged on five days after their surgeries.

The primary outcome was total perioperative calculated blood loss after MOWHTO, according to the formulae reported by a previous study, [26] and was calculated based on the difference between the patient’s total blood volume as reported by Nadler et al. [27] and the difference in hemoglobin (Hb) between before and after surgery. First, the patient’s blood volume was calculated using the following formula:

PBV (L) = k1 × height [m]^3^ + k2 × weight (kg) + k3 (1)

where k1 = 0.3669, k2 = 0.03219, and k3 = 0.6041 for men and k1 = 0.3561, k2 = 0.03308, and k3 = 0.1833 for women.

Next, the loss of Hb was measured using the following formula:

Hb loss = blood volume × (Hbi − Hbe) × 10 dL/L+ Hbt(2)

where Hb loss is the amount of Hb lost by three days postoperation; Hbi (g/L) is the Hb concentration prior to MOWHTO; Hbe (g/L) is the Hb concentration at three days after surgery; and Hbt (g/L) is the amount of Hb transfused. In this case, if a transfusion was not performed, then Hbt was omitted.

Finally, the blood loss (mL) was determined using the following formula:

Blood loss (mL) = 100 mL/dL × Hb loss/Hbi.(3)

The total drain amount was measured on the first, second, and third days after surgery. In addition, the total amount of drainage over three days was also calculated. The Hb values were investigated before surgery and at one, two, and five days and two and six weeks after surgery.

Secondary outcomes included self-reported pain severity at rest, with walking, at its worst and least, at nighttime, and over an average of 24 h according to a 10-point visual analog scale (VAS). Patient-reported outcomes were measured using the EuroQol-5 Dimension (EQ-5D) questionnaire [28], which is a commonly used and validated measurement tool for assessing health-related quality of life. It was developed by the EuroQol group and consists of five parts: mobility, self-care, daily activities, pain/discomfort, and anxiety/depression. Each category is measured according to three ratings: no problems, some problems, and extreme problems [28]. The postoperative allogeneic transfusions rate was also evaluated. Criteria for the transfusion of blood products included a Hb level of less than 7.0 g/dL or a Hb level of less than 8.0 g/dL with clinical signs of symptomatic anemia, such as unexplained tachycardia, dyspnea, or hypotension [29,30]. In addition, all wound-related measures, including hematoma, wound dehiscence, oozing, suture granuloma, and superficial wound infection, during the follow-up period were included as wound complications [31]. Regardless of DVT symptoms, routine computed tomography (CT) venography (Revolution CT; GE Medical Systems, Waukesha, WI, USA) was performed five days postoperatively to detect DVT in all patients.

### 2.2. Statistical Analysis

An a priori sample-size calculation was conducted based on previously published results in patients who underwent MOWHTO [14]. The estimated blood loss was set to 502.4 mL in the TXA group and 882.7 mL in the control group, with standard deviation values defined as 294.9 mL in the TXA group and 482.0 mL in the control group [14]. The level of significance (α = 0.05 (both sides)) was defined and the power was maintained at 90% with a type 2 error (β) = 0.10. The estimated sample size was 26 patients for each group; thus, 35 patients were needed per group based on an estimate of 25% follow-up loss. Differences between two groups for continuous variables were measured using an independent t-test or the Mann–Whitney U test. Differences were measured using the chi-squared test or Fisher’s exact test for categorical variables. The level of significance was set at *p* < 0.05. All analyses were implemented with the use of the Statistical Package for the Social Sciences version 24.0 software program (IBM Corporation, Armonk, NY, USA)

## 3. Results

**Figure 2:**
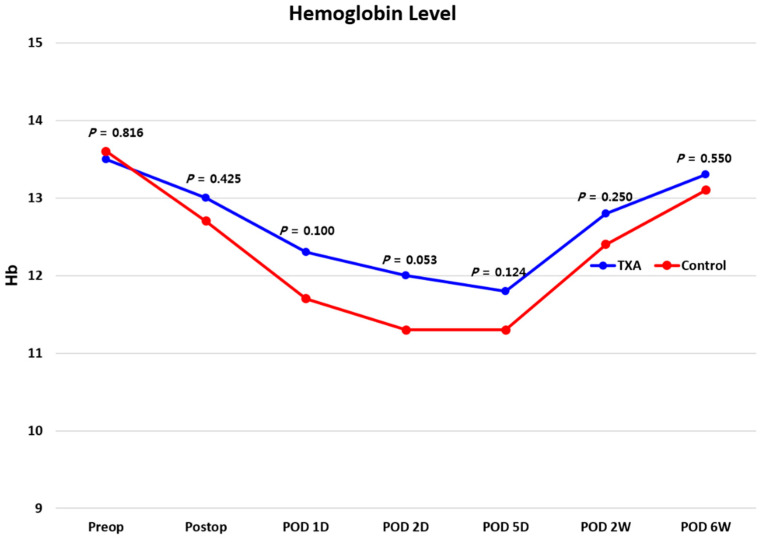
Comparison of Hemoglobin values between two groups.

There was no difference in demographic data between the two groups (Table 1). The total amount of blood loss after surgery was 470.9 mL in the TXA group and 739.3 mL in the control group, showing a significant difference between the two groups (*p* < 0.05). No difference in the Hb level between the two groups between before and after surgery during follow-up existed (Figure 2). However, the differences between the preoperative Hb level and the Hb level immediately after surgery and on the first, second, and fifth days after surgery were significantly lower in the TXA group than in the control group (all *p* < 0.05). The total amount of drain output was 170.8 mL in the TXA group and 328.0 mL in the control group, with a significant difference (*p* < 0.05) (Table 2).

**Figure 3:**
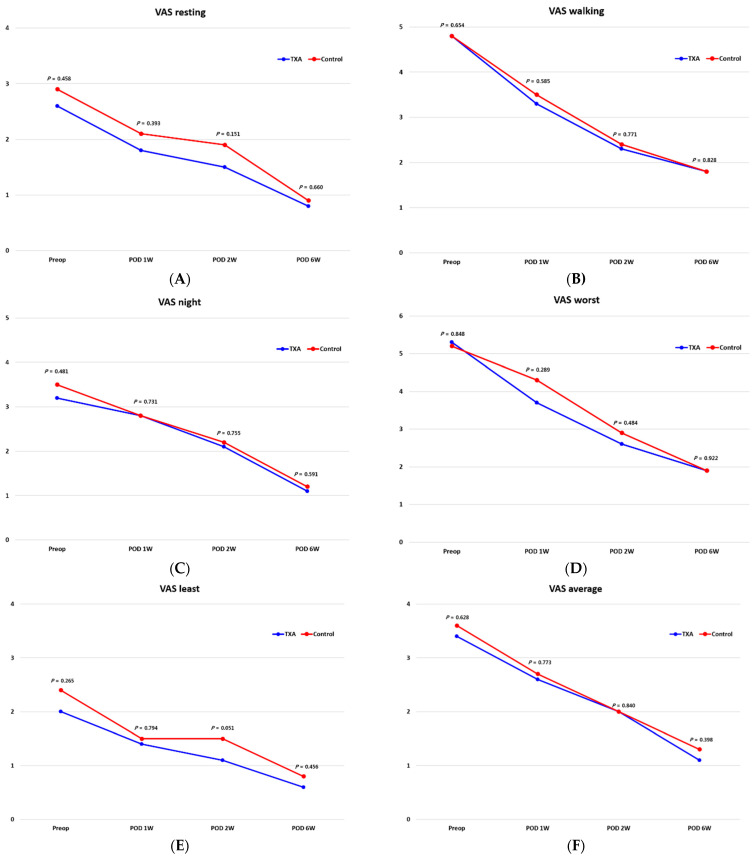
Pain VAS: Rest (**A**), walking (**B**), night (**C**), worst (**D**), least (**E**), and average (**F**).

**Figure 4:**
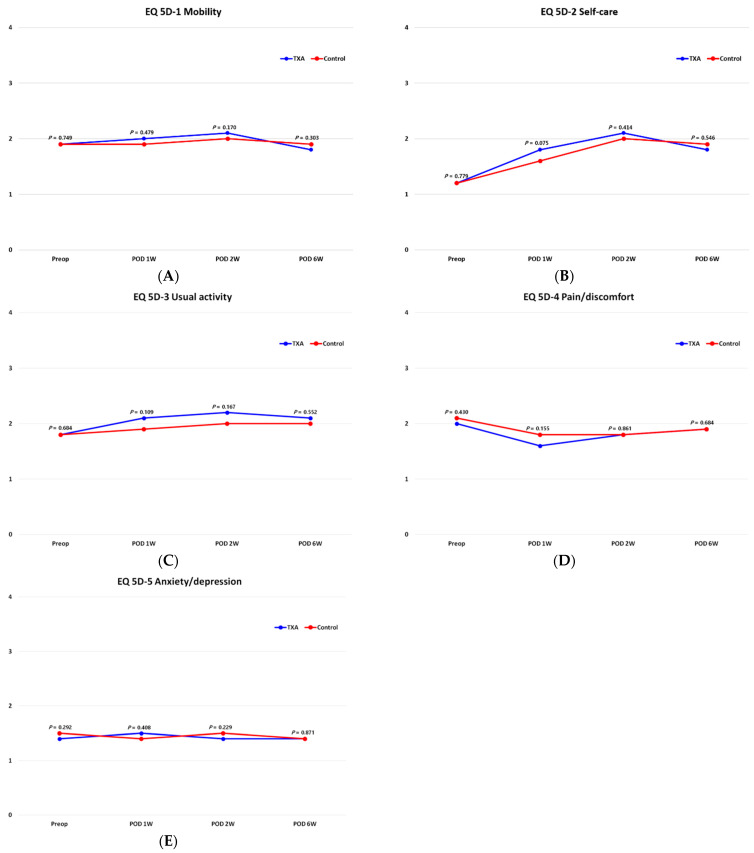
Quality of life assessment using the EQ-5D. Mobility (**A**), self-care (**B**), usual activity (**C**), pain/discomfort (**D**), and anxiety/depression (**E**).

There was no difference in resting, walking, nighttime, worst, least, and average pain VAS scores between the two groups before surgery or until six weeks after surgery (*p* < 0.05) (Figure 3). The preoperative EQ-5D scores revealed no difference between the two groups in all items, and the same results were apparent at one, two, and six weeks after surgery (Figure 4).

During the follow-up period, two cases of wound complications occurred in the TXA group and three cases occurred in the control group (*p* > 0.05). There was one instance of wound dehiscence in each group and additional suturing was performed. In one case of the TXA group, antibiotics were additionally administered due to superficial wound infection. In one case of the control group, additional antibiotics administration, and compression dressing were performed due to wound redness and swelling. In both groups, except for one additional suture case in each group, no additional measures were taken due to a wound complication or infection (Table 3).

There were no clinically symptomatic DVT findings in both groups. However, as a result of DVT CT imaging on the fifth day after surgery, 23 cases (62.2%) of DVT were found to have occurred in the TXA group and 15 cases (41.7%) of DVT were found to have occurred in the control group. There was no significant difference in the DVT rate between the two groups, although more cases were reported in the TXA group (*p* = 0.103). There were 37 cases of DVT in the calf vein, two cases in popliteal vein, and two cased in femoral vein. DVT was found in the calf and femoral veins in one patient and in the calf and popliteal veins in two patients. Asymptomatic pulmonary embolism (PE) was found in a total of 7 patients. Patients with DVT and PE were referred to the vascular surgeon and cardiologist to take anticoagulant medication if necessary and to follow up with the appropriate department. No transfusion was performed in either group.

## 4. Discussion

The most important finding of this study is that patients who received IV TXA experienced significantly less total blood loss after MOWHTO than those in the control group. There were no differences between the two groups in terms of pain, quality of life, rate of transfusion, wound complications, and DVT incidence.

Research on the use of IV TXA in TKA has been actively conducted, and, in fact, a consensus has been established for the use of IV TXA in TKA [18,32,33,34]. However, research on the use of IV TXA when implementing MOWHTO remains sparse [5,7,8,14]. We are aware of only one prospective RCT that compared TXA and placebo therapy in patients undergoing HTO. One RCT and three retrospective studies did not clearly provide the basis for the use of IV TXA in patients undergoing MOWHTO [5,7,8,14]. The abovementioned RCT lacked data for the safety of DVT occurrence [7] (Table 4).

In MOWHTO, all studies on TXA showed that the total blood loss, drainage, and Hb reduction were significantly less in the group that used TXA than in the group that did not [7,8,10,14]. Ni et al. [7]. reported that estimated total blood losses were 478 mL in the TXA group and 835 mL in the control group. Kim et al. [14] also reported losses of 883 mL in the TXA group and 502 mL in the control group, with a significant difference. Han et al. [10] also observed a significant difference between the loss of 372 mL with topical TXA and that of 635 mL in the control group. Our study demonstrated similar results. In fact, the use of IV TXA had sufficient advantages in terms of total blood loss after MOWHTO. This fact can be sufficiently confirmed given the reduction in total drainage amount and the change in Hb level (Table 4).

However, the purpose of reducing such total blood loss is to reduce transfusion and quickly return patients to a state of well-being. As compared with arthroplasty patients, the patients in this study were younger and did not require transfusion with or without TXA following MOWHTO. The criterion for transfusion was that Hb was less than 7 g/dL, but there were no cases in which the postoperative Hb level was less than 7 g/dL in either the TXA or control group after surgery. Although transfusion is sometimes performed in MOWHTO, it is true that the ratio is less than that during arthroplasty. Ni et al. [7] and Kim et al. [14] reported no transfusion cases in the TXA group and one transfusion case in the control group. Palanisamy et al. [8] reported no cases of transfusion in both the TXA and control groups. These results are the same as ours. In the case of MOWHTO, there is a possibility of needing a transfusion after surgery but, since the rate is so small, it is difficult to expect the rate of transfusion will be reduced by lessening the amount of blood loss with IV TXA (Table 4).

There was no difference between the two groups in the rate of wound complications in this study, similarly to as confirmed in previous studies. In the study by Ni et al. [7], no wound complications occurred in the TXA group, while cases of hematoma and superficial wound infections occurred in the control group. Similarly, Palanisamy et al. [8] reported that only participants in the control group experienced hematoma and infection. However, neither study identified any significant difference in wound complications between the two groups [7,8]. Of course, this result may have been due to an insufficient number of participants to show differences in wound complications, but, in our study, there was also no difference in the rate of wound complications between the two groups (Table 4).

Reduction of bleeding at the surgical site also helps to reduce pain at the surgical site and may affect recovery [5,8,9,10,14]. This study evaluated whether reducing blood loss through the use of TXA is beneficial to the clinical aspect. However, VAS and EQ-5D scores did not exhibit any significant differences between the two groups until six weeks after surgery. In fact, few studies have compared clinical outcomes to investigate the effects of TXA on MOWHTO. Palanisamy et al. [8] only compared VAS scores retrospectively between the two groups and reported that, although the TXA group actually had less pain, there was no significant difference in such relative to the control group. It seems that the reduction in blood loss caused by TXA did not appear to be effective enough to make a difference in clinical manifestations. Moreover, in the case of MOWHTO, partial weight-bearing using crutches was conducted for up to six weeks after surgery, so it is difficult to clearly compare these pain and QOL scores with those recorded after full weight-bearing.

IV TXA is a safe drug that does not increase the risk of DVT as much while also reducing blood loss, as demonstrated in previous studies. However, studies with accurate evaluations of DVT when IV TXA was used in patients who underwent MOWHTO are limited [5,7,8,10,14]. Most studies have contended that no DVT existed when patients did not have DVT-related symptoms [5,7,8,10,14]. In the study by Ni et al., the DVT occurrence was evaluated by sonography, and there was no DVT case in both groups. Palanisamy et al. [8] determined the presence or absence of DVT based on patients’ symptoms, reporting that there was no symptomatic DVT in either group. Kim et al. [14] judged DVT by DVT CT imaging and stated that there was no DVT in either group. However, these results were different from our findings; in this study, roughly half of patients in both groups developed asymptomatic DVT, although none of these patients had DVT-related symptoms. Moreover, although there was no difference in the rate of DVT between the two groups, the TXA group experienced a higher incidence. DVT prophylaxis was not performed in the patients in this study, and these findings suggest that DVT prophylaxis might have to be considered in MOWHTO. As a result, the use of IV TXA in MOWHTO is stable for symptomatic DVT, but it is difficult to regard it as a stable drug given the confirmation of asymptomatic DVT by CT (Table 4).

There were several limitations to this study. First, most patients in our study were women. Women have a high proportion of osteoarthritis of the knee joint [35,36,37,38,39,40,41]. Second, the follow-up period was six weeks, which might be too short to clearly grasp the effects of IV TXA. Third, a clinical trial with a small sample number has limitations for identifying differences in secondary outcomes, including wound complications and DVT. A large cohort study is needed to clearly confirm these differences. Fourth, ours was a study involving two surgeons who perform surgery in the same way. Although reproducible results can be obtained, limitations might exist in terms of generalization. Finally, the method of measuring estimated blood loss is indirect, and, for accuracy, measurements of Hb before surgery and those recorded serially after surgery would be optimal. However, in this study, preoperative Hb level was measured two weeks before surgery on average, and could have differed from the Hb level immediately before surgery.

## 5. Conclusions

In patients undergoing MOWHTO, IV TXA was efficacious in reducing the total amount of blood loss without additional benefits in the areas of wound complications, transfusion, clinical outcome, and DVT incidence.

## Figures and Tables

**Table 1:** Baseline patient characteristics.

Characteristics	TXA Group (*n* = 37)	Control Group (*n* = 36)	*p*-Value
Age, mean (SD), y	54.9 (9.2)	55.3 (7.0)	0.852
Sex			0.844
Male	10 (27.0%)	9 (25.0%)	
Female	27 (73.0%)	27 (75.0%)	
Body mass index, mean (SD)	26.1 (3.1)	26.0 (5.3)	0.96
Comorbidities			
Hypertension	15 (40.5%)	14 (38.9%)	0.885
Diabetes	5 (13.5%)	5 (13.9%)	0.963
Brain	0 (0%)	0 (0%)	1
Thyroid	2 (5.6%)	0 (0%)	0.146
Kidney	0 (0%)	1 (2.8%)	0.307
Lung	1 (2.7%)	0 (0%)	0.321
Smoking	6 (16.2%)	1 (2.8%)	0.051
Alcohol	2 (5.4%)	2 (5.6%)	0.978
ASA score			0.254
1	10 (27.0%)	14 (38.9%)	
2	26 (70.3%)	19 (52.8%)	
3	1 (2.7%)	3 (8.3%)	
KL grade			0.832
2	15 (41.7%)	14 (38.9%)	
3	20 (55.6%)	20 (55.6%)	
4	1 (2.8%)	2 (5.6%)	
Preoperative mechanical axis (MA, °)	Varus 6.9 (2.6)	Varus 6.7 (3.6)	0.754
Preoperative weight bearing line (%)	17.0 (11.8)	19.2 (14.9)	0.477
Postoperative MA (°)	Valgus 2.8 (2.6)	Valgus 2.6 (1.9)	0.637
Postoperative WBL (%)	61.6 (7.0)	60.9 (9.0)	0.745
Preoperative hemoglobin (d/dL)	13.5 (1.3)	13.6 (1.8)	0.904
Preoperative Hematocrit (%)	40.9 (3.6)	40.7 (5.2)	0.848
Preoperative blood platelet (×109/L)	264.8 (58.8)	243.7 (69.5)	0.166
Preoperative activated partial thromboplastin time (s)	27.0 (2.3)	27.4 (2.9)	0.475

**Table 2:** Drain output, total blood loss, and Hb value decrease in the study groups.

	TXA Group (*n* = 37)	Control Group (*n* = 36)	*p*-Value
Drain output (mL) 0–24 h	87.5 (101.1)	225.7 (136.2)	<0.001
Drain output (mL) 24–72 h	83.3 (49.7)	102.4 (104.7)	0.326
Total drain output (mL)	170.8 (122.2)	328.0 (194.0)	<0.001
Total blood loss (mL)	470.9 (236.0)	739.3 (314.4)	<0.001
Hemoglobin decrease			
Preoperation to immediately postoperation	0.5 (0.6)	0.9 (0.7)	0.008
Preoperation to 1 day postoperation	1.2 (0.8)	1.9 (0.8)	0.001
Preoperation to 2 days postoperation	1.5 (0.7)	2.4 (1.0)	<0.001
Preoperation to 5 days postoperation	1.7 (0.9)	2.4 (1.1)	0.007

**Table 3:** Intergroup comparison of wound complications.

	TXA Group (*n* = 37)	Control Group (*n* = 36)	*p*-Value
Wound complication			
Wound dehiscence	1 (2.7%)	1 (2.8%)	0.984
Hematoma aspiration	0 (0%)	0 (0%)	1.000
Drainage occurring after day 5 postoperation	0 (0%)	1 (2.8%)	0.307
Suture granuloma	0 (0%)	0 (0%)	1.000
Additional antibiotics for redness	0 (0%)	1 (2.8%)	0.984
Superficial surgical-site infection	1 (2.7%)	0 (0%)	0.321

**Table 4:** Comparison of tranexamic acid related studies in medial opening wedge high tibial osteotomy.

Authors	Group	No. of Patients	Total Blood Loss (mL)	Total Drainage Volume (mL)	Hemoglobin Reduction (Pre to Postoperative 2 Days) (g/dL)	Transfusion	Deep Vein Thrombosis	Wound Complication
Suh et al. [5]	TXA	15		246.0	1.7	NR	NR	0 (0%)
(2018)	Control	15		377.2	1.2	NR	NR	1 (6.7%)
Ni et al. [7]	TXA	50	477.9	282.3	2.6	0 (0%)	0 (0%)	0 (0%)
(2020)	Control	50	834.6	413.2	3.3	1 (2%)	0 (0%)	2 (4%)
Palanisamy et al. [8]	TXA	66	372.0	315.0	1.3	0 (0%)	0 (0%)	0 (0%)
(2018)	Control	86	635.0	537.0	2.2	0 (0%)	0 (0%)	2 (2.3%)
Kim et al. [14]	TXA	77	502.4	269.3	2.3	0 (0%)	0 (0%)	NR
(2018)	Control	75	882.7	330.4	3.2	2 (2.6%)	0 (0%)	NR
Our study (2021)	TXA	37	470.9	170.8	1.5	0 (0%)	23 (62.2%)	2 (5.4%)
	Control	36	739.3	328.0	2.4	0 (0%)	15 (41.7%)	3 (8.3%)

NR: not reported.

## Data Availability

Data collected for this study, including individual patient data, will not be made available.

## References

[1] Kim H.J., Shin J.Y., Lee H.J., Park K.H., Jung C.H., Kyung H.S. (2020). Can medial stability be preserved after open wedge high tibial osteotomy?. Knee Surg. Relat. Res..

[2] Na Y.G., Lee B.K., Choi J.U., Lee B.H., Sim J.A. (2021). Change of joint-line convergence angle should be considered for accurate alignment correction in high tibial osteotomy. Knee Surg. Relat. Res..

[3] Ogbemudia A.O., Bafor A., West-Osemwengie L. (2012). Reactionary haemorrhage reduction with adrenaline infiltration in proximal tibial osteotomy: A randomized clinical study of safety and efficacy. Arch. Orthop. Trauma Surg..

[4] Park S.H., Jung K.H., Chang S.W., Jang S.M., Park K.B. (2020). Trends in knee surgery research in the official journal of the Korean Knee Society during the period 1999–2018: A bibliometric review. Knee Surg. Relat. Res..

[5] Suh D.W., Kyung B.S., Han S.B., Cheong K., Lee W.H. (2018). Efficacy of Tranexamic Acid for Hemostasis in Patients Undergoing High Tibial Osteotomy. J. Knee Surg..

[6] Pape D., Dueck K., Haag M., Lorbach O., Seil R., Madry H. (2013). Wedge volume and osteotomy surface depend on surgical technique for high tibial osteotomy. Knee Surg. Sports Traumatol. Arthrosc..

[7] Ni J., Liu J., Zhang J., Jiang J., Dang X., Shi Z. (2020). Tranexamic acid is beneficial for blood management of high tibial osteotomy: A randomized controlled study. Arch. Orthop. Trauma Surg..

[8] Palanisamy J.V., Das S., Moon K.H., Kim D.H., Kim T.K. (2018). Intravenous Tranexamic Acid Reduces Postoperative Blood Loss After High Tibial Osteotomy. Clin. Orthop. Relat. Res..

[9] Duivenvoorden T., van Diggele P., Reijman M., Bos P.K., van Egmond J., Bierma-Zeinstra S.M.A., Verhaar J.A.N. (2017). Adverse events and survival after closing- and opening-wedge high tibial osteotomy: A comparative study of 412 patients. Knee Surg. Sports Traumatol. Arthrosc..

[10] Han S.B., In Y., Oh K.J., Song K.Y., Yun S.T., Jang K.M. (2019). Complications Associated With Medial Opening-Wedge High Tibial Osteotomy Using a Locking Plate: A Multicenter Study. J. Arthroplast..

[11] Seo S.S., Kim O.G., Seo J.H., Kim D.H., Kim Y.G., Lee I.S. (2016). Complications and Short-Term Outcomes of Medial Opening Wedge High Tibial Osteotomy Using a Locking Plate for Medial Osteoarthritis of the Knee. Knee Surg. Relat. Res..

[12] Woodacre T., Ricketts M., Evans J.T., Pavlou G., Schranz P., Hockings M., Toms A. (2016). Complications associated with opening wedge high tibial osteotomy—A review of the literature and of 15 years of experience. Knee.

[13] Hernigou P., Giber D., Dubory A., Auregan J.C. (2020). Safety of simultaneous versus staged bilateral opening-wedge high tibial osteotomy with locked plate and immediate weight bearing. Int. Orthop..

[14] Kim K.I., Kim H.J., Kim G.B., Bae S.H. (2018). Tranexamic acid is effective for blood management in open-wedge high tibial osteotomy. Orthop. Traumatol. Surg. Res..

[15] Bjerke-Kroll B.T., Sculco P.K., McLawhorn A.S., Christ A.B., Gladnick B.P., Mayman D.J. (2014). The increased total cost associated with post-operative drains in total hip and knee arthroplasty. J. Arthroplast..

[16] Hill G.E., Frawley W.H., Griffith K.E., Forestner J.E., Minei J.P. (2003). Allogeneic blood transfusion increases the risk of postoperative bacterial infection: A meta-analysis. J. Trauma.

[17] Eubanks J.D. (2010). Antifibrinolytics in major orthopaedic surgery. J. Am. Acad. Orthop. Surg..

[18] Gillette B.P., Kremers H.M., Duncan C.M., Smith H.M., Trousdale R.T., Pagnano M.W., Sierra R.J. (2013). Economic impact of tranexamic acid in healthy patients undergoing primary total hip and knee arthroplasty. J. Arthroplast..

[19] Henry D.A., Carless P.A., Moxey A.J., O’Connell D., Stokes B.J., Fergusson D.A., Ker K. (2011). Anti-fibrinolytic use for minimising perioperative allogeneic blood transfusion. Cochrane Database Syst. Rev..

[20] Kagoma Y.K., Crowther M.A., Douketis J., Bhandari M., Eikelboom J., Lim W. (2009). Use of antifibrinolytic therapy to reduce transfusion in patients undergoing orthopedic surgery: A systematic review of randomized trials. Thromb. Res..

[21] Zufferey P.J., Miquet M., Quenet S., Martin P., Adam P., Albaladejo P., Mismetti P., Molliex S. (2010). Tranexamic acid in hip fracture surgery: A randomized controlled trial. Br. J. Anaesth..

[22] Lee D.C., Byun S.J. (2012). High tibial osteotomy. Knee Surg. Relat. Res..

[23] Fujisawa Y., Masuhara K., Shiomi S. (1979). The effect of high tibial osteotomy on osteoarthritis of the knee. An arthroscopic study of 54 knee joints. Orthop. Clin. N. Am..

[24] Kim M.S., Son J.M., Koh I.J., Bahk J.H., In Y. (2017). Intraoperative adjustment of alignment under valgus stress reduces outliers in patients undergoing medial opening-wedge high tibial osteotomy. Arch. Orthop. Trauma Surg..

[25] Lobenhoffer P., Agneskirchner J.D. (2003). Improvements in surgical technique of valgus high tibial osteotomy. Knee Surg. Sports Traumatol. Arthrosc..

[26] Konig G., Hamlin B.R., Waters J.H. (2013). Topical tranexamic acid reduces blood loss and transfusion rates in total hip and total knee arthroplasty. J. Arthroplast..

[27] Nadler S.B., Hidalgo J.H., Bloch T. (1962). Prediction of blood volume in normal human adults. Surgery.

[28] Bilbao A., Martín-Fernández J., Arenaza J., García I., Tomás-García N., Trujillo-Martín E., García-Perez L. (2017). Validation of the EQ-5D-5L in patients with hip or knee osteoarthritis. Value Health.

[29] Carson J.L., Guyatt G., Heddle N.M., Grossman B.J., Cohn C.S., Fung M.K., Gernsheimer T., Holcomb J.B., Kaplan L.J., Katz L.M. (2016). Clinical Practice Guidelines From the AABB: Red Blood Cell Transfusion Thresholds and Storage. JAMA.

[30] Viberg B., Gundtoft P.H., Schønnemann J., Pedersen L., Andersen L.R., Titlestad K., Madsen C.F., Lauritsen J., Overgaard S. (2018). Introduction of national guidelines for restrictive blood transfusion threshold for hip fracture patients—A consecutive cohort study based on complete follow-up in national databases. J. Orthop. Surg. Res..

[31] Kim M.S., Koh I.J., Lee S.Y., In Y. (2018). Central sensitization is a risk factor for wound complications after primary total knee arthroplasty. Knee Surg. Sports Traumatol. Arthrosc..

[32] Bradley K.E., Ryan S.P., Penrose C.T., Grant S.A., Wellman S.S., Attarian D.E., Green C.L., Risoli T., Bolognesi M.P. (2019). Tranexamic acid or epsilon-aminocaproic acid in total joint arthroplasty? A randomized controlled trial. Bone Jt. J..

[33] Stowers M.D.J., Aoina J., Vane A., Poutawera V., Hill A.G., Munro J.T. (2017). Tranexamic Acid in Knee Surgery Study-A Multicentered, Randomized, Controlled Trial. J. Arthroplast..

[34] Tsukada S., Kurosaka K., Nishino M., Maeda T., Hirasawa N., Matsue Y. (2020). Intraoperative Intravenous and Intra-Articular Plus Postoperative Intravenous Tranexamic Acid in Total Knee Arthroplasty: A Placebo-Controlled Randomized Controlled Trial. J. Bone Jt. Surg. Am..

[35] Choi Y., Koo J., Moon S.W., Yang Y., Son J. (2020). Long-term Follow-up of Patellar Nonresurfacing in Total Knee Arthroplasty. Clin. Orthop. Surg..

[36] Chon J., Jeon T., Yoon J., Jung D., An C.-H. (2019). Influence of Patellar Tilt Angle in Merchant View on Postoperative Range of Motion in Posterior Cruciate Ligament-Substituting Fixed-Bearing Total Knee Arthroplasty. Clin. Orthop. Surg..

[37] Jang S., Shin W.C., Song M.K., Han H.-S., Lee M.C., Ro D.H. (2021). Which orally administered antithrombotic agent is most effective for preventing venous thromboembolism after total knee arthroplasty? A propensity score-matching analysis. Knee Surg. Relat. Res..

[38] Lee H.I., Park D., Cho J. (2018). Clinical and Radiological Results with Second-Look Arthroscopic Findings after Open Wedge High Tibial Osteotomy without Arthroscopic Procedures for Medial Meniscal Root Tears. Knee Surg. Relat. Res..

[39] Lee O.S., Lee E.S., Lee Y.S. (2019). Disparity between Preoperative Target Correction Amount and Postoperative Correction Amount in Open Wedge High Tibial Osteotomy. Knee Surg. Relat. Res..

[40] Noh J.H., Kim N.Y., Song K.I. (2021). Intraoperative patellar maltracking and postoperative radiographic patellar malalignment were more frequent in cases of complete medial collateral ligament release in cruciate-retaining total knee arthroplasty. Knee Surg. Relat. Res..

[41] Yoo J.H., Oh H.C., Park S.H., Kim J.K., Kim S.H. (2018). Does Obesity Affect Clinical and Radiological Outcomes in Minimally Invasive Total Knee Arthroplasty? Minimum 5-Year Follow-up of Minimally Invasive TKA in Obese Patients. Clin. Orthop. Surg..

